# Mood Disorders among Older Adults Participating in Individual and Group Active Environments: “Me” versus “Us,” or Both?

**DOI:** 10.1155/2012/727983

**Published:** 2012-07-17

**Authors:** Rachael C. Stone, Brad A. Meisner, Joseph Baker

**Affiliations:** ^1^School of Kinesiology & Health Science, York University, Toronto, ON, Canada M3J 1P3; ^2^Department of Psychology, Ryerson University, Toronto, ON, Canada M5B 2K3

## Abstract

Involvement in physical activity is associated with improved mental health including better social skills, coping mechanisms, and lower rates of depression. However, evidence on whether group or individual active environments better facilitate these benefits remains inconsistent. This cross-sectional cohort study examined the mental health reports of older adults (aged 50+) in relation to participation in group or individual active environments. Logistic multivariate regression analyses were conducted on the Canadian Community Health Survey (cycle 4.1, 2007-2008, *n* = 44, 057). Results illustrated that those active in both group and individual environments were 59% less likely to have a mood disorder than those who were not participating in either (*P* < 0.001). Also, those active in both environments were 31% less likely to have a mood disorder than those active in an individual environment (*P* < 0.001). Participating in only group or only individual environments had a similar effect compared to individuals not active in any environments for reducing rates of reported mood disorders (22% and 28%, resp.). However, the findings related to only group environments were not significant. These findings reveal that participating in both group and individual physical activities may have important implications for maintaining older adults' mental health status.

## 1. Introduction

Older adults in Canada are the fastest growing cohort of the population [1], a trend echoed in other industrialized nations [2]. Currently, over 35% of the Canadian population is comprised of individuals aged 50 years and above, a number expected to rise as the baby boom generation progresses further into older age [2, 3]. Research has concluded that older individuals who are living longer often do so with a reduced quality of life and a greater disease/disability burden [4]. With these outcomes in mind, a growing field of research has begun to focus on how to facilitate “successful aging” amongst the aging population.

A vital component of many successful aging models pertains to maintaining one's psychological and mental health [5, 6]. 

Mood disorders are a growing health concern for an aging population. For example, the percentage of Canadians reporting a diagnosed mood disorder rose from 5.3% in 2003 to 6.3% in 2009; 43% of those reporting a mood disorder in 2009 were 50 years of age and greater [7, 8]. A major mood disorder is an umbrella term for a range of depressive and manic disorders, and their variants. Depressive disorders are marked by experiencing negative affect, loss of interest in usual enjoyable activities, irritability, and catastrophizing mentalities for at least two weeks. Dysthymia is a more severe and chronic form of depression [9]. On the other hand, manic disorders, which include mania and bipolar disorders, tend to persist for at least a week and are characterized by distinct fluctuations in one's disposition (e.g., being unusually talkative, loud, egomaniacal, paranoid, and reckless) [9]. Mood disorders are most commonly treated with pharmacological agents and/or psychological treatments [9]. Recently, more cost-effective treatments have begun to be explored, including physical activity [10, 11]. 

Physical activity has been found to lessen the negative and maladaptive symptoms of mood disorders on biochemical, physiological, and psychological levels [11]. Interestingly, evidence from clinical studies has shown that the psychological benefits associated with participating in exercise and physical activities are comparable to those found with standard forms of psychotherapy [12, 13]. Moreover, there is a considerable amount of literature demonstrating the important psychological benefits that consistent exercise participation can offer the aging population including improvements in cognitive function, positive affect, self-efficacy, social skills, cohesion, networking, and engagement, life and sexual satisfaction, as well as reduced incidences of psychological chronic conditions including mood disorders such as depression and schizophrenia [14–23]. 

The “active environment” describes whether one participates in exercise within a group or an individual setting, and it facilitates mental health benefits [13, 15, 24–28]. However, much of the previous research on exercise environments has focused on how individual *versus* group physical activities affect adoption of, and adherence to, exercise or physical activity among the older adult population. The evidence on whether older individuals prefer exercising in a group or individual activities has been inconsistent and somewhat contradictory. For instance, Beauchamp et al. (2007) found that 68% of adults aged 50 or more preferred participating alone in a one-year aerobic program [13]. In contrast, Fox et al. (2007) noted that European older adults preferred exercising in a group environment during a one-year aerobic program [26]. Although these two studies are contradictory in terms of older adult preferences for exercise, both studies highlight similar mental health benefits from physical activity participation in group and individual environments. 

 In addition to active environment preferences, previous research has shown inconsistent findings in relation to maintaining exercise participation within a specific mode of an active environment (i.e., group versus individual). Researchers, (e.g., [15, 24, 25, 28]) have highlighted that participating in group exercise produces superior attendance rates among older adults aged 50+ compared to exercising alone. This effect appears to be adjunct to perceptions of group cohesion and belonging as participants felt strongly that they were part of a team, producing an average attendance rate of 85% for group exercise programs. However, in contrast, King et al. (1993) found that adherence rates were slightly higher when participants aged 50+ were placed in an individual, home-based exercise program compared to a group-based program. The success was attributed to participants perceiving greater internal locus of control over their health [27]. 

Research regarding the accruement of mental health benefits among older adults has also been mixed in regards to the active environment. King et al. (1993) found no significant differences in positive mental health outcomes between group and individual exercise environments during a one-year randomized control trial of aerobic exercise, and it was claimed that group exercise was unnecessary as older adults could receive similar benefits from more convenient forms of individual exercise [27]. In contrast, Brawley et al.'s (2000) nine-month randomized control trial of group versus individual exercise environment interventions found that self-efficacy, social skills, and general mental health were significantly greater among those in the group active environment than the individual one [24].

It is important to note that these previous data are based on structured exercise single-blind interventions rather than voluntary or natural participation in less regimented physical activity or sport. Recent discussions of the role physical activity and sport in promoting healthy aging, (e.g., [29, 30]) have advocated that sport and physical activity produce improvements in health and functioning above and beyond those obtained via standardized exercise routines. 

Considering the potential mental health concerns for the aging population and the contradictory findings in previous research in this area of research, the current study examined a mental health outcome among Canadian older adults who were physically active in group and/or individual active environments. The primary objective of this analysis was to examine whether these modes of active environments are associated with mental health. An important secondary goal was to determine the prevalence of these two active environments among older adults in Canada, an objective that contributes to the research on exercise and physical activity preferences among older adults.

## 2. Methods

### 2.1. Participants

Cross-sectional data from the Canadian Community Health Survey (CCHS, cycle 4.1) were used. Information collected in the CCHS considers diseases, health, lifestyle, and social conditions, which was collected from December to January of 2007 and 2008. The CCHS contains specific data on sport participation and physical activity behaviours. Cycle 4.1 of the CCHS has a sample size of 131,061 voluntary participants, both male and female, aged 12 and greater. Volunteers were randomly recruited from all provinces and territories. Participants completed the survey via computer-assisted interviews, over the telephone, or their personal computers. The CCHS is praised for having a high nation-wide response rate (86%) and for being representative of the Canadian population. Participants in the current study were a subsample of the overall dataset, as some questionnaire content was optional (i.e., exercise habits, perceived life stress, and drinking/smoking behaviours) [3]. As a result, analyses were first limited to respondents who had a complete set of data for the variables under investigation. The sample was further limited to individuals 50 years of age or greater in correspondence with much of the existing research in this research area. The final sample included 44,057 participants. Ethics approval for the current study was granted by the York University Research Ethics Committee.

### 2.2. Measurements

#### 2.2.1. Outcome Variable

Mental health was defined by the presence of a mood disorder. The question in the CCHS assessed whether an individual had been diagnosed with depression, bipolar disorder, mania, or dysthymia by a healthcare professional in the past 12 months (yes/no). This question was mandatory for all CCHS participants.

### 2.3. Main Predictor Variable

#### 2.3.1. Active Environment

This variable was divided into four categories based on self-reported leisure physical activity and sport participation: those participating in only group active environments, those participating in only individual active environments, those participating simultaneously in both environments, and those participating in neither. This variable, which is not available in the CCHS, was calculated using Microsoft Excel 2003 to identify the respondents in each category. Group active environments were defined as physical activities or sports that *require* interactions among individuals when participating. The activities meeting this criterion in the CCHS were ice hockey, baseball/softball, volleyball, basketball, and soccer. Individual active environments were defined as activities or sports that *do not require* interactions among individuals when participating. The activities meeting this criterion in the CCHS were walking, gardening, swimming, bicycling, jogging, golf, and tennis/racquetball. Classification of this variable was captured by summing the total number of participants who responded “yes” to participating in one or more group or individual activities. Furthermore, those participating in both environments simultaneously responded “yes” to one or more activities from group *and *individual categories while others responded “no” to both sets of activities were not participating in either of the specified active environments. Resulting from this classification were four possible categorical responses for the active environments variable: (1) those participating in only group active environments, (2) those participating in only individual active environments, (3) those participating simultaneously in both active environments, and (4) those participating in neither active environment. These specific activities were chosen as the present study aimed to assess purposeful physical activity respondents completed in their leisure time. As such, the present study does not assess incidental physical activity (i.e., transportation, occupational, or house work). 

### 2.4. Covariates

Based on previous research [31–35], a number of covariates were included in the analyses to minimized their confounding effects on the key associations under investigation. First, sociodemographic variables, such as age, sex, and marital status were included. Age was classified in the CCHS in five-year groups, beginning from 50–54 years to 75–79 years with a final group of those aged 80 and greater. Sex was reported as “male” or “female,” and marital status was defined as being “married,” “common-law,” “divorced/widowed,” or “single.” Second, given that negative health behaviours such as alcohol consumption and smoking are linked with decreases in physical activity [36], they were also included as health behavioural covariates. Regarding the alcohol consumption variable, six categories represented the frequency of binge drinking (i.e., consuming five or more drinks in one sitting): “never,” “less than once per month,” “once per month,” “2-3 times per month,” “once per week,” and “more than once per week.” Three categories represented smoking status: “daily,” “occasional,” and “not at all.” Moreover, considering sport participation intensity can vary considerably and may moderate the likelihood of having or developing a mood disorder, the participant's intensity of physical activity was controlled for in the analyses using the Physical Activity Index variable available within the CCHS based on energy expenditure. Energy expenditure was calculated using average metabolic equivalent values (a measure of intensity) and self-reported frequencies and durations of participants' physical activity endeavours. This variable categorizes respondents as being “active,” “moderately active,” or “inactive” in their leisure time activities based on the total daily energy expenditure values over the last three months (kcal/kg of body weight/day). “Active” was defined as burning 3 or more kcal/kg/day, “moderately active” as burning between 1.5 and 2.9 kcal/kg/day, and “inactive” as burning between 0 and 1.4 kcal/kg/day [3, 26]. Finally, given that the dependent variable relates to the mental health domain, a variable on psychological stress was also included as a covariate to control for this variable's potential influence on the relationships examined. Five categories represented individuals' perceptions of life stress: “not at all,” “not very,” “a bit,” “quite a bit,” and “extremely.” 

### 2.5. Analyses

A bivariate logistic regression analysis was used to examine the relationships between the active environments and the presence of a mood disorder (Model A). Subsequently, a multivariate logistic regression analysis was estimated to examine the aforementioned relationship, controlling for the sociodemographic, health behavioural, and psychological stress covariates (Model B). Given that this was a subsample of the overall CCHS sampling strategy due to the optional content within the CCHS included in this analysis, population weights were not applied. Effect sizes were reported as odds ratios (OR), and the significance levels were represented by 95% confidence interval (CI) for all analyses. Analyses were conducted using SPSS version 19.0 software.

## 3. Results

**Figure 1:**
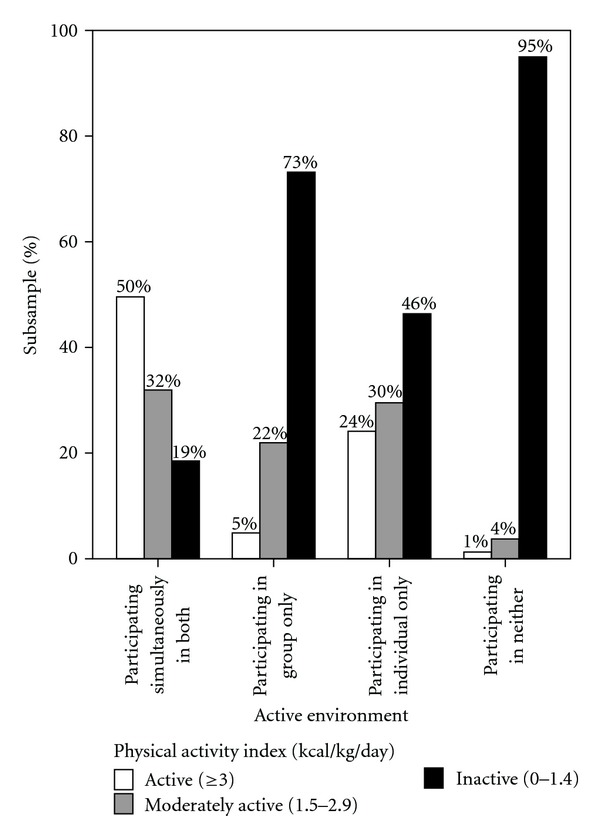
Percentage of participants participating in active environments stratified by daily energy expenditure levels.

Table 1 displays descriptive information of the sample. The majority of the older adults were aged 50–64 years (57.6%), female (54.4%), married (55.4%), nonbinge drinkers (71.8%), nonsmokers (81.4%), inactive (52.2%), and perceived their lives to be “a bit” stressed (35.2%). Seven percent of the sample reported the presence of a diagnosed mood disorder. The vast majority of older adults in this sample were participating in individual active environments only (83.2%). Participation in group environments only was reported by an extremely small proportion of participants in this sample (0.1%) while a relatively larger proportion of these older adults participated in both group and individual active environments (3.1%). In comparison, many more reported not participating in either group or individual active environments (13.6%). It should be noted that although 13.6% did not participate in either of the two active environments, 52.2% of the participants were classified as “inactive” according to the Physical Activity Index (i.e., expending less than or equal to 1.4 kcal/kg/day, Table 1). This finding is a result of the fact that a participant can be involved in the specified active environments, but not at a high enough intensity level to be considered “active” or “moderately active.” It is interesting to note, however, that 73.0% of participants active in group environments were classified as “inactive” (Figure 1). On the other hand, 1.0% and 4.0% of the participants who are not active in either environment classified as “active” and “moderately active,” respectively (Figure 1), suggesting that these individuals expended energy outside of the leisure activities included in the current study.

The bivariate (Model A) and multivariate (Model B) associations between active environment and the likelihood of having a mood disorder are presented in Table 2. At the bivariate level, older adults participating in both group and individual active environments were 59.0% less likely to have a mood disorder than those who are not participating in any group or individual active environments (OR = 0.41, CI = 0.31–0.55). In addition, those participating in both types of active environments were 31.0% and 37.0% less likely to have a mood disorder than those participating in individual-only or group-only active environments, respectively (CIs = 0.65–0.79 and 0.24–2.5). These findings echo data presented in Figure 1 as 50.0% of participants engaged in both types of active environments were physically active at the most intense level, compared to all other types of active environments. Those participating in individual active environments only were 28.0% less likely to have a mood disorder than those who did not participate in either active environments (OR = 0.72, CI = 0.65–0.79). There were no statistically significant differences between those participating in group active environments and nonactive environment participants regarding the presence of a mood disorder (CI = 0.24–2.5). After adjusting for the covariates (i.e., age, sex, marital status, binge drinking, smoking, physical activity index, and life stress), comparable results were found. More specifically, only small decreases were observed in the effect sizes regarding the likelihood of having a mood disorder in the adjusted model, and all of the significance values remained.

## 4. Discussion

The present investigation found that most of the older adult Canadian population preferred to participate in individual active environments compared to group active environments. This finding is supported by previous research that describes older adults' preferences for individual activities, possibly to maintain their functional independence [13]. The result that older adults active in individual environments experience a decreased likelihood of reporting a mood disorder may be due to increased levels of self-efficacy that many older adults experience when participating in these activities via an internally focused locus of control of one's health [13, 15, 24–28]. This effect would allow older individuals to attribute their physical and psychological health improvements to their own actions rather than to external forces. Other evidence suggests that participation in individual active environments may promote the development of intrapersonal coping mechanisms to counter the psychological stresses, allowing individuals to adjust the stressors, maladaptive behaviours, and emotions associated with mood disorders [9, 37]. 

Regarding the results on whether participating in group and/or individual active environments had an influence on older adult's mental health outcomes, it was found that exercising solely in individual active environments decreased the likelihood of having a mood disorder, while participating in group environments had no significant effect. The present study does not question whether group active environments are beneficial to health in general; however, it does suggest that the benefits older adults derive from this mode of active environments *alone* may not pertain to mental health or the prevalence of the mood disorders included in the current investigation. Despite the fact that participating in group-only active environments was not statistically associated with the odds of having a mood disorder, older adults who participated in both group and individual active environments were the least likely to have a mood disorder. This finding suggests the beneficial nature of participating in individual activities in conjunction with group activities.

The notion that individual and group activities function together (i.e., via an additive effect) to promote mental health in later life is supported by previous research. Studies have shown similar benefits associated with each environment upon mental health outcomes. For example, improved self-efficacy is a benefit of participating in individual active environments among older adults [24], and this association is also observed among those active in group environments [15, 16]. It is also possible that individuals who participated in both group and individual active environments had a greater involvement in physical activity and sport, therefore, having a greater energy expenditure per day than those active in either environment alone (Figure 1). This possibility may explain why this particular group is the least likely to have a mood disorder (Table 2). Much like previous research [38–41], the present findings suggest that the more active environments one participates in at a moderate-vigorous intensity level, the better symptoms of mood disorders are attenuated. However, it is important to note that the relationship between active environment and the likelihood of having a mood disorder remained significant even after controlling for the physical activity index, a general measure of exercise intensity (Table 2; Model B).

### 4.1. Limitations

The CCHS contains self-reported data and may be prone to social desirability bias. As well, the cross-sectional nature of these data prevents an analysis of the cause and effect directionality of the relationships under investigation. In addition, due to the fact that several variables were included in the CCHS as optional content, the available subsample comprised of only 30 percent of the overall CCHS sample size, which may limit the generalizability of these findings. Also, regarding the classification of the active environments variable, it is possible that participants participated in individual activities as part of a group environment. For instance, swimming was coded as an individual activity because it does not *require* interactions with others to participate; however, participants could belong to a swimming group. Unfortunately, the structure of the CCHS does not provide these distinctive qualities of these two active environments.

### 4.2. Future Research

There is a need for continued research in the field of active environments and mental health outcomes for older adults, not only to address the limitations of the current study, but to discern the relative, and perhaps additive, contributions that different modes of active environments have at promoting successful aging in the mental health domain. This field of research would benefit from objective measures and designs, such as randomized control trials, that include individual and group activities to establish a clear cause-and-effect relationship between active environments and mental health in later life. As many of the previous studies have represented active environments as separate entities in their research designs, future studies should include experimental groups that assess group and individual active environments both separately and in conjunction with one another. Furthermore, as much of the current research on active environments focuses on structured exercise regimes, physical activity and sport-related research is lacking, yet such studies are required to validate the findings of the present study.

## 5. Conclusion

The present study revealed findings consistent with previous literature on mental health outcomes and active environments. Using a large dataset of older adults, this study supports the notion that participation in individual active environments can have benefits on one's mental health. However, this investigation has shown that participation in individual active environments along with group active environments may be even more effective at fostering positive mental health outcomes in later life. These findings suggest that engagement in multimodal active environments may be an important protective strategy against mood disorders among older adults. Overall, these findings have interesting and important implications on promoting successful aging among older Canadian adults [42]. These results should facilitate further research on the topic of active environments to clarify its relationship to mental health and other domains of health as well.

## Figures and Tables

**Table 1:** Sample descriptive statistics for all variables in the analyses (*N* = 44,057).

Variable and category	*n*	(%)
Mood disorders		
Yes	3073	7.0
No	40,984	93.0
Active environment		
Group only	41	0.1
Individual only	36,659	83.2
Group + individual	1356	3.1
Neither	6001	13.6
Age (years)		
50–54	8776	19.9
55–59	8767	19.9
60–64	7850	17.8
65–69	5949	13.5
70–74	4809	10.9
75–79	3866	8.8
80 years and greater	4040	9.2
Sex		
Males	20,095	45.6
Females	23,962	54.4
Marital status		
Married	24,406	55.4
Common law	2315	5.3
Widowed/divorced	13,720	31.1
Single	3616	8.2
Physical activity index		
Active (≥3.0 kcal/kg/day)	9597	21.8
Moderately active (1.5–2.9 kcal/kg/day)	11,482	26.1
Inactive (0–1.4 kcal/kg/day)	22,978	52.2
Frequency of binge drinking		
Never	31,652	71.8
Less than once/month	6881	15.6
Once/month	1727	3.9
2-3 times/month	1342	3.0
Once/week	1342	3.0
More than once/week	1113	2.5
Type of smoker		
Daily	7034	16.0
Occasional	1161	2.6
Never	35,862	81.4
Perceived life stress		
Not at all	8612	19.5
Not very	13,011	29.5
A bit	15,508	35.2
Quite a bit	5806	13.2
Extremely	1120	2.5

**Table 2:** Results of logistic regression analysis for the relationship between active environments and likelihood of having a mood disorder (*N* = 44,057).

Variable	Model A: OR (95% CI)	Model B: OR (95% CI)
Group only	0.78 (0.24, 2.5)^‡^	0.83 (0.25, 2.8)^‡^
Individual only	0.72 (0.65, 0.79)	0.85 (0.77, 0.95)
Group + individual	0.41 (0.31, 0.55)	0.55 (0.41, 0.74)
Neither	1.00 (referent)	1.00 (referent)

All *P* ≤ 0.01, except ^‡^ *P* ≥ 0.05 (not significant), Model A: bivariate, unadjusted associations. Model B: multivariate, adjusted associations independent of age, sex, marital status, binge drinking, type of smoker, physical activity index, and perceived life stress.
